# Research progress of OTUD7B: from structural function and disease mechanisms to clinical translation

**DOI:** 10.3389/fimmu.2026.1850848

**Published:** 2026-06-09

**Authors:** Qingsong Wang, Shuqiong Xu, Lihui Wen, Juan Meng, Xianmin Wang, Tongyong Luo, Renjiu Luo, Zhujun Li, Dan Lin, Jun Yin

**Affiliations:** 1 Department of Pediatrics, West China Hospital Sichuan University Jintang Hospital, Jintang First People’s Hospital, Chengdu, Sichuan, China; 2 Pediatric Cardiology Center, Sichuan Provincial Women’s and Children’s Hospital/The Affiliated Women’s and Children’s Hospital of Chengdu Medical College, Chengdu, Sichuan, China; 3 General Department, Guancang Community Health Service Center, Chengdu, Sichuan, China; 4 Department of Ultrasound, West China Hospital Sichuan University Jintang Hospital/Jintang First People’s Hospital, Chengdu, Sichuan, China

**Keywords:** deubiquitinase, OTUD7B, prognostic biomarker, signaling pathways, tumor microenvironment

## Abstract

OTUD7B (Cezanne), a deubiquitinase of the Ovarian Tumor (OTU) domain family, regulates the ubiquitin-proteasome system and plays a role in maintaining cellular homeostasis. Its dysfunction is linked to the pathogenesis of multiple human diseases. In neoplastic diseases, OTUD7B is overexpressed in several solid tumors, including non-small cell lung cancer (NSCLC), gastric cancer, breast cancer, and pancreatic cancer. It promotes tumor cell proliferation, invasion, metastasis, and chemoresistance by stabilizing oncoproteins such as YAP1, ERα, and β-catenin/LEF1. This stabilization activates signaling pathways including NF-κB, Wnt/β-catenin, and Notch. However, its function is context-dependent. In diffuse large B-cell lymphoma (DLBCL), OTUD7B stabilizes TRAF3 to inhibit the non-canonical NF-κB pathway. In hepatocellular carcinoma (HCC), it deubiquitinates and stabilizes p53 to induce apoptosis. In both contexts, high expression correlates with favorable prognosis. In non-neoplastic diseases, OTUD7B also plays a dual role. In experimental autoimmune encephalomyelitis (EAE) models, it protects neurons by deubiquitinating RIPK1 and stabilizing GFAP. This action suppresses inflammation and promotes glial scar formation. Conversely, in pathological cardiac hypertrophy, its role is model-dependent. It protects against ferroptosis by stabilizing HNF4α in pressure-overload models. Under neurohormonal stimulation, it promotes hypertrophy by deubiquitinating SERCA2a and disrupting calcium homeostasis. These findings indicate that OTUD7B is a context-specific regulator. Its expression levels are associated with prognosis in multiple diseases. It shows potential as a diagnostic and prognostic biomarker and as a therapeutic target for specific conditions. This review systematically summarizes the molecular characteristics, expression regulation, physiological functions, and mechanisms of OTUD7B in neoplastic and non-neoplastic diseases. It also discusses the prospects and challenges of its clinical translation.

## 1 Introduction

Deubiquitinases (DUBs) govern protein fate and signal transduction within the ubiquitin-proteasome system. Among them, the ovarian tumor (OTU) domain-containing proteases display divergent roles in immunity and cancer. OTUD7B (Cezanne), an A20-like OTU member, exhibits significant functional plasticity: it drives malignant progression in most solid tumors yet suppresses tumor growth in diffuse large B-cell lymphoma and hepatocellular carcinoma. This context-dependent dichotomy raises three unresolved questions. First, what determines whether OTUD7B functions as an oncogene or a tumor suppressor? Second, how does a single deubiquitinase achieve signaling specificity across diverse substrates without systemic dysregulation? Third, what are the principal barriers to selective therapeutic targeting given its indispensable physiological roles? Existing literature has cataloged individual substrates and disease associations, yet an integrative analysis of the mechanistic roots of this functional plasticity—and a focused assessment of translational feasibility—remains lacking. This review systematically addresses these gaps by delineating the molecular architecture, context-dependent pathological functions, and clinical translational prospects of OTUD7B, aiming to advance understanding from mechanistic description toward precision intervention.

## 2 Biological characteristics of OTUD7B

### 2.1 Molecular features

**Figure 1:**
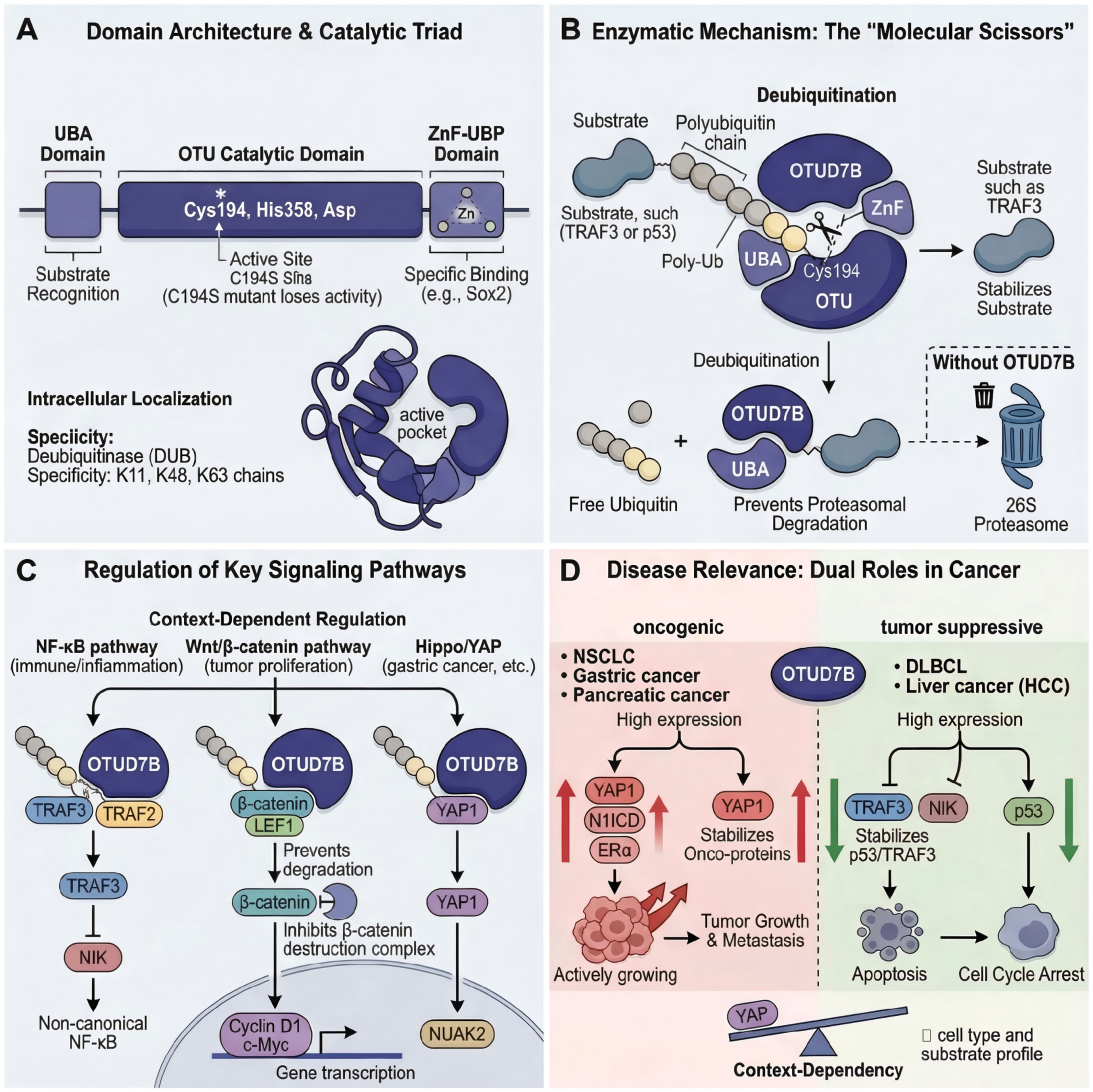
Molecular structure, enzymatic mechanism, and signaling regulation of OTUD7B. **(A)** Domain organization showing the UBA, OTU catalytic (C194S), and ZnF domains. **(B)** Enzymatic mechanism: OTUD7B cleaves K11/K63-linked polyubiquitin chains to stabilize substrates (e.g., TRAF3, p53). **(C)** Regulation of key pathways (NF-κB, Wnt/β-catenin, Hippo/YAP). **(D)** Dual roles in cancer: oncogenic (e.g., NSCLC) vs. tumor-suppressive (e.g., HCC).

OTUD7B (Ovarian Tumor Domain Containing 7B), also known as Cezanne, belongs to the Ovarian Tumor domain-containing protease (OTU) superfamily and is a core deubiquitinase. The gene is located on human chromosome 15q24.1. It is primarily distributed in the cytoplasm but can undergo nuclear translocation or co-localize with membrane receptor complexes under specific signal stimuli (e.g., the β-catenin complex in the Wnt pathway or at the immunological synapse) (1). As a key regulator of the ubiquitin-proteasome system (UPS), OTUD7B maintains the homeostasis of critical intracellular molecules by hydrolyzing ubiquitin chains on target proteins, thereby blocking substrate degradation or altering their signaling status (2) (**Figures 1A–D**).

Structural biological studies confirm that OTUD7B possesses a highly modular domain architecture, which determines its substrate recognition specificity and catalytic efficiency:

Catalytic Core (OTU Domain): Located in the middle of the protein, it contains a classical catalytic triad composed of cysteine (Cys194), histidine (His358), and aspartic acid. Cys194 is the core of universal catalytic activity; the C194S mutation results in a complete loss of enzymatic activity. His358 plays a key role in specific substrate recognition; the H358R mutant retains partial basal enzymatic activity but specifically loses the ability to mediate the deubiquitination of the transcription factor Sox2 (3, 4).

N-terminal Ubiquitin-Binding Domain (UBA): Responsible for recognizing and binding substrates containing specific ubiquitin chain modifications. This domain is essential for physical interaction with substrates such as TRAF2 and LEF1; deletion of the UBA directly blocks binding and weakens signal regulation capabilities (5, 6).

C-terminal Zinc Finger Domain (ZF): Exhibits extremely high substrate specificity, dedicated to directly recognizing and binding the transcription factor Sox2, reflecting the fine division of labor among different domains in substrate screening (3).

Enzymatically, OTUD7B demonstrates broad ubiquitin chain selectivity, capable of targeting Lys11 (K11), Lys48 (K48), and Lys63 (K63)-linked ubiquitin chains for hydrolysis (7). This diversity allows it to flexibly regulate substrate fate based on ubiquitin chain type: primarily inhibiting proteasome-mediated degradation by removing K48/K11 chains, or modulating signal transduction functions by modifying K63 chains. Although the full-length isoform is currently the main reported variant, bioinformatic analysis suggests the existence of multiple splice variants that may regulate functional specificity by lacking specific domains.

### 2.2 Mechanisms of expression regulation

**Figure 2:**
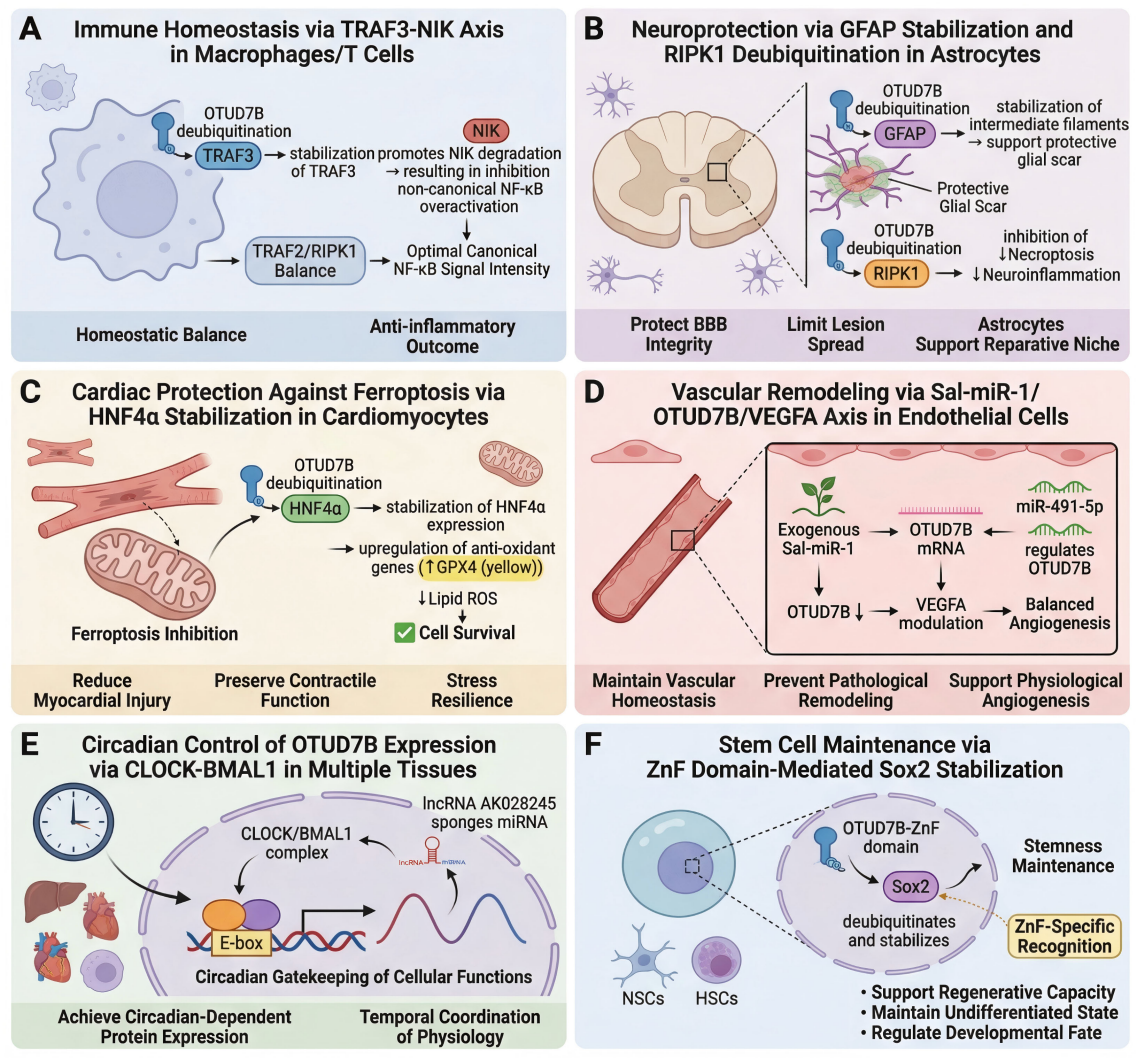
Physiological roles of OTUD7B across systems. **(A)** Immune homeostasis: regulation of the TRAF3-NIK axis. **(B)** Neuroprotection: stabilization of GFAP/RIPK1 in astrocytes. **(C)** Cardioprotection: inhibition of ferroptosis via HNF4α-GPX4. **(D)** Vascular remodeling: regulation by Sal-miR-1/VEGFA. **(E)** Circadian regulation by CLOCK-BMAL1. **(F)** Stem cell maintenance: ZnF-mediated stabilization of Sox2.

OTUD7B expression is controlled by a multi-layered regulatory network. This network integrates circadian clock signals, non-coding RNA interference, and epigenetic modifications. Such multi-level control ensures accurate spatiotemporal responses under diverse physiological conditions (**Figures 2A–F**).

At the circadian rhythm level, OTUD7B is a direct downstream target gene of the core clock transcription factor CLOCK (Clock-controlled gene). Its promoter region contains E-box elements. Under physiological conditions, CLOCK protein indirectly relieves the inhibition of OTUD7B by suppressing the transcription of the long non-coding RNA AK028245, constructing a positive regulatory axis of “CLOCK → lncRNA AK028245↓ → OTUD7B↑”. This results in significant 24-hour circadian fluctuations in mRNA and protein levels, a process crucial for maintaining immune system homeostasis and temporal precision (8).

In post-transcriptional regulation, the microRNA miR-491-5p plays a key role by directly binding to the 3’UTR region of OTUD7B mRNA, negatively regulating its expression by hindering translation (9). Notably, cross-species regulation also exists: exogenous plant small RNAs derived from *Salvia miltiorrhiza* (Sal-miR-1 and Sal-miR-3) can specifically recognize two independent sites in the OTUD7B mRNA 3’UTR region, further suppressing its expression at the post-transcriptional level (2).

At the epigenetic level, the involvement of the N6-methyladenosine (m6A) methyltransferase METTL14 suggests that RNA methylation modification is a key factor in its fine-tuned regulation (10). Additionally, under inflammatory stimulation, a negative feedback loop involving positive regulation by NF-κB may also participate.

This complex regulatory network ultimately shapes the significant tissue specificity and disease-dependent expression characteristics of OTUD7B. Under physiological conditions, it is highly abundant in cells with high metabolic or immune activity, such as astrocytes, retinal photoreceptors, and dendritic cells (DCs), matching its functional demand for maintaining cellular homeostasis and immune balance (11, 12). In pathological conditions, its expression shows bidirectional dynamic changes: in cardiac hypertrophy models, the infarct border zone of myocardial infarction rats, and hypoxia-treated cardiac fibroblasts, OTUD7B mRNA and protein levels are significantly downregulated, which may be closely related to the loss of cardioprotective functions and anti-fibrotic capabilities (13). Conversely, in various malignant tumors such as gastric and breast cancer, and in progressive astrocytes in disease models like multiple sclerosis, expression levels are markedly elevated, suggesting a potential dual role in remodeling the tumor microenvironment or pathological processes of neuroinflammation (11).

### 2.3 Physiological functions

As a core cellular stabilizer, OTUD7B extensively participates in physiological processes including signal transduction, cell cycle regulation, immune balance, tissue fibrosis control, and targeted protection. Through its unique domain combination (OTU, UBA, ZF) and a broad spectrum of substrate interactions (including TRAF2/3, Sox2, GβL, ERα, LSD1, N1ICD, GFAP, Smac, YAP1, VEGFA, RIPK1, β-catenin, LEF1, KLF4, etc.), it constructs a vast regulatory network covering immune homeostasis, metabolic regulation, embryonic development, cell cycle, tissue protection, and anti-fibrosis, serving as a key molecular hub for maintaining organismal health and stability.

#### 2.3.1 Context-dependent dual regulation of immune homeostasis

OTUD7B is a key regulator of the nuclear factor kappa-light-chain-enhancer of activated B cells (NF-κB) signaling pathway, with functions exhibiting significant context dependency. In the non-canonical NF-κB pathway, OTUD7B acts as a molecular brake by deubiquitinating and stabilizing the negative regulator TRAF3, preventing its degradation. This blocks the accumulation of NF-κB-Inducing Kinase (NIK) and spontaneous activation of the non-canonical pathway, maintaining the resting state of immune cells such as macrophages and avoiding excessive inflammatory responses (4, 8). Conversely, upon dendritic cell (DC) activation or acute inflammatory stimulation, OTUD7B switches to a promoter of the canonical pathway: it stabilizes TRAF2 and promotes its K63 ubiquitination, thereby activating receptor-interacting serine/threonine-protein kinase 1 (RIPK1) and driving moderate activation of NF-κB and mitogen-activated protein kinase (MAPK) pathways. This function is crucial for preventing tumor necrosis factor (TNF)-induced apoptosis and ensuring effective priming of pathogen-specific CD8+ T cells (5). Additionally, OTUD7B can cooperate with the homologous protein A20 to perform sequential K63 and K48 deubiquitination modifications on RIPK1, limiting the spread of inflammatory storms (11).

#### 2.3.2 Core regulation of cell growth, metabolism, and developmental signals

In cell growth and metabolic regulation, OTUD7B is an essential component for the complete assembly of the mammalian target of rapamycin complex 2 (mTORC2). It maintains the stability of the key subunit GβL by removing its K63 ubiquitination modification, thereby promoting Akt phosphorylation and activation, which directly determines cell growth rate, glucose metabolism, and lipid synthesis (14). In the fields of development and stem cell regulation, OTUD7B activates Notch signaling by forming a ternary complex (interacting with RHBDL2 and the Notch1 intracellular domain/N1ICD) to remove ubiquitin chains from N1ICD (1). Simultaneously, it relies on its specific C-terminal ZF domain to deubiquitinate and stabilize Sox2, maintaining the stemness of neural progenitor cells (NPCs) (3). Furthermore, OTUD7B enhances Wnt (Wingless-related integration site) signal transduction by stabilizing β-catenin and LEF1 and intervenes in the RAS signaling cascade by interacting with RASGRF1 and PLCE1, broadly influencing cell proliferation and differentiation (1).

#### 2.3.3 Cell cycle, epigenetics, and hormone signaling networks

OTUD7B ensures the smooth transition of the cell cycle from G1 to S phase by stabilizing the histone demethylase LSD1 and regulating the periodic fluctuation of anaphase-promoting complex/cyclosome (APC/C) substrates (15, 16). In hormone signal regulation, it directly binds to the activation function 1 (AF1) domain of the estrogen receptor alpha (ERα), removing its K11/K48 ubiquitin chains to enhance hormone transduction, tightly coupling epigenetic modification with the hormone response network (17). Additionally, OTUD7B can directly interact with and deubiquitinate Kruppel-like factor 4 (KLF4), inhibiting its proteasomal degradation, thereby deeply participating in the regulation of phenotypic switching in vascular smooth muscle cells (VSMCs) (2).

#### 2.3.4 Tissue-specific protection and cell fate decisions

In nervous system protection, OTUD7B maintains blood-brain barrier integrity by K48 deubiquitination and stabilization of glial fibrillary acidic protein (GFAP), a key cytoskeletal protein in astrocytes (11), while also protecting retinal cone cells from light damage (12). In cardiovascular system protection, it alleviates pressure overload-induced cardiac hypertrophy by protecting mitochondrial function in cardiomyocytes and inhibiting ferroptosis (18). In cardiac fibroblasts, OTUD7B exhibits potent anti-cardiac fibrosis properties by regulating the FAK-ERK/P38 MAPK signaling axis to inhibit cell activation and extracellular matrix synthesis (e.g., α-SMA, Type I collagen), independently of the TGF-β/Smad pathway (13). In cell life-and-death decisions, OTUD7B exhibits complex dual effects: on one hand, it promotes cell survival by binding the pro-apoptotic protein Smac to inhibit its K48 ubiquitination degradation (19), or by stabilizing YAP1 to activate the YAP1/NUAK2 axis (20); on the other hand, it can directly interact with and stabilize VEGFA, promoting physiological angiogenesis (9).

In summary, through precise multi-pathway and multi-tissue regulation, OTUD7B has become a key molecular node connecting cellular homeostasis and pathological processes. Its functional abnormalities may lead to various pathological states such as immune imbalance, metabolic disorders, tissue injury, and tumor progression.

### 2.4 Comparative substrate specificity and functional crosstalk with OTU paralogs

Understanding the functional boundaries of OTUD7B requires contextualization within the OTU domain-containing deubiquitinase family. OTUB1, OTUD1, and OTUD7B share a conserved cysteine protease fold, yet their catalytic properties and biological outcomes diverge substantially. OTUB1 preferentially hydrolyzes Lys48 (K48)-linked polyubiquitin chains and employs a non-canonical mechanism—blocking E2-ubiquitin conjugation—to stabilize substrates such as programmed death-ligand 1 (PD-L1) (21). OTUD1 has been implicated in the regulation of Smurf1 and mitochondrial antiviral-signaling protein (MAVS), though its ubiquitin chain-type preference remains less characterized (22). In contrast, OTUD7B exhibits broad chain selectivity, cleaving Lys11 (K11), K48, and Lys63 (K63) linkages through its catalytic triad (Cys194-His358-Asp), with the C-terminal zinc finger domain conferring additional substrate specificity (4, 9). This enzymatic promiscuity positions OTUD7B as a signaling integrator rather than a linear pathway component.

These catalytic differences translate into distinct immune regulatory strategies. OTUB1 promotes immune evasion by stabilizing PD-L1 and suppressing AKT-dependent T cell activation (21). OTUD1 attenuates antiviral immunity by deubiquitinating Smurf1, thereby accelerating MAVS degradation and dampening type I interferon production (22). OTUD7B exhibits context-dependent duality: in dendritic cells, it stabilizes TRAF2 to promote canonical NF-κB signaling and CD8+ T cell priming (23); in B cells, it stabilizes TRAF3 to restrain non-canonical NF-κB activation (4). Notably, OTUD7B further distinguishes itself by negatively regulating antiviral immunity through selective autophagic degradation of interferon regulatory factor 3 (IRF3)—a mechanism unique among OTU members (24).

The mechanistic divergence between these paralogs carries significant therapeutic implications. OTUB1 stabilizes PD-L1 through non-catalytic ubiquitin transfer blockade, rendering traditional catalytic-site inhibitors ineffective against this oncogenic function (21). In contrast, OTUD7B relies predominantly on its catalytic activity to stabilize substrates, which provides a rationale for catalytic inhibitors (4)] (25, 26). However, the high conservation of the OTU catalytic domain across family members poses a selectivity crisis: off-target inhibition of OTUB1 could paradoxically enhance PD-L1 stability and promote immune evasion (21). Consequently, family-selective drug design must distinguish catalytic (OTUD7B) from non-catalytic (OTUB1) molecular modalities or alternatively exploit PROteolysis TArgeting Chimera (PROTAC)-mediated degradation strategies to achieve specificity (27, 28).

## 3 Roles and molecular mechanisms of OTUD7B in various diseases

**Figure 3:**
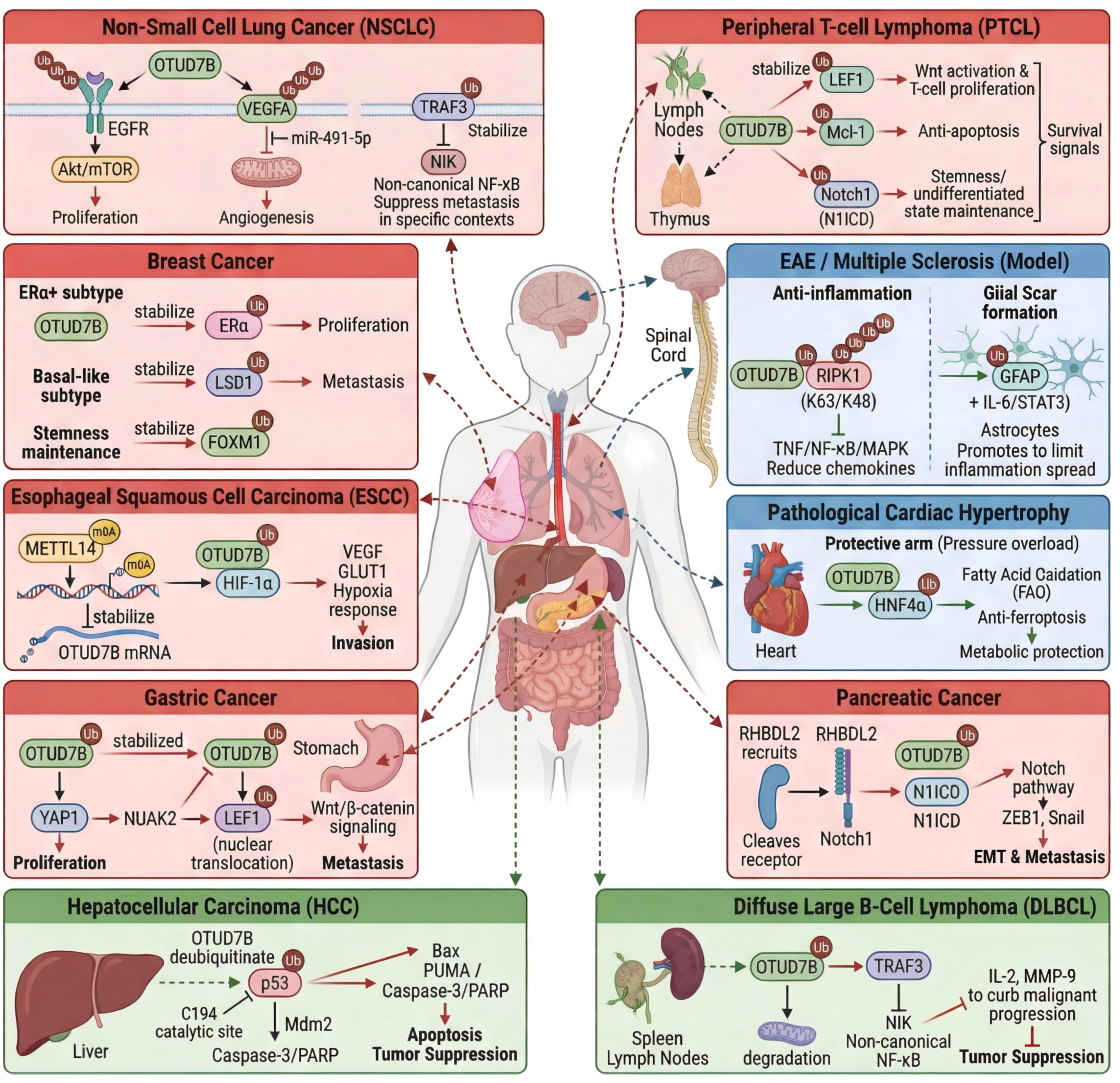
Disease-specific molecular mechanisms of OTUD7B. Schematic illustration of OTUD7B’s context-dependent functions across various malignancies and non-malignant diseases. Oncogenic roles: In cancers such as NSCLC, Breast, Gastric, ESCC, PTCL, and PDAC, OTUD7B stabilizes oncoproteins (e.g., EGFR, ERα, YAP1, N1ICD) to promote proliferation, metastasis, and stemness. Tumor-suppressive roles: Conversely, in HCC and DLBCL, OTUD7B stabilizes tumor suppressors (p53, TRAF3) or inhibits malignant progression. Protective roles in non-malignant diseases: OTUD7B exerts protective effects in Heart Failure (via HNF4α) and EAE (via RIPK1) by limiting tissue damage and inflammation. Red boxes indicate pathogenic contexts; blue/green boxes indicate protective contexts.

As a key regulator of intracellular ubiquitination modification, abnormal expression or dysfunction of OTUD7B is closely related to the occurrence and development of various human diseases (**Table 1**). From the proliferation and metastasis of malignant tumors to inflammatory degeneration of the nervous system, and pathological remodeling of the cardiovascular system, OTUD7B exhibits complex and diverse regulatory networks in different pathological contexts. The following details the specific roles and molecular mechanisms of OTUD7B in various diseases (**Figure 3**, red panels indicate oncogenic contexts; blue panels indicate tumor-suppressive contexts; green panels indicate protective non-neoplastic contexts).

**Table 1:** Summary of OTUD7B expression profiles, core mechanisms and evidence in multiple diseases.

Disease category	Disease	OTUD7B expression/functional alteration	Primary substrate	Core mechanisms	Key experimental/clinical evidence	Literature ID
Neoplastic Diseases	Non-small cell lung cancer	Significantly upregulated; poor prognostic factor	EGFR, VEGFA, TRAF3	1. Promotes angiogenesis via AKT/VEGF pathway. 2. Regulates miR-491-5p/VEGFA axis. 3. Deubiquitinates and stabilizes TRAF3, inhibiting LCL161-induced invasion.	High expression correlates with advanced stage, metastasis, shorter OS; promotes proliferation, migration, invasion *in vitro* and *in vivo*.	(4, 7, 9)
	Pancreatic cancer	Highly expressed; drives malignant progression	N1ICD (Notch1 intracellular domain)	Synergizes with RHBDL2 to deubiquitinate and stabilize N1ICD, sustaining Notch signaling.	Knockdown or catalytic inactivation suppresses proliferation, migration, invasion; depends on OTUD7B-RHBDL2-N1ICD axis.	(1)
	Gastric cancer	Significantly upregulated; independent poor prognostic factor	YAP1, LEF1, β-catenin	1. Deubiquitinates and stabilizes YAP1, activating YAP1/NUAK2 axis. 2. Interacts with LEF1 to activate Wnt/β-catenin pathway and promote metastasis.	High expression correlates with high grade, lymphatic invasion, poor prognosis; knockdown suppresses malignant phenotypes.	(20, 50)
	Breast cancer	Subtype-specific function and prognosis	ERα, LSD1, FOXM1	1. ERα+ subtype: stabilizes ERα → proliferation and endocrine resistance. 2. Basal-like subtype: stabilizes LSD1 → metastasis. 3. Stabilizes FOXM1 → maintains cancer stemness.	High expression correlates with shorter OS in ERα+ patients; silencing reverses drug resistance, inhibits metastasis and stemness.	(15, 17, 34)
	Peripheral T-cell lymphoma	Downregulated; poor prognostic factor	LEF1, Mcl-1	Loss of function causes non-canonical NF-κB (NIK) overactivation, promoting survival and proliferation.	Low OTUD7B predicts poor prognosis independently.	(60)
	Diffuse large B-cell lymphoma	Highly expressed; favorable prognostic factor	TRAF3	Deubiquitinates and stabilizes TRAF3 → promotes NIK degradation → suppresses non-canonical NF-κB.	High expression correlates with longer OS; upregulation enhances sensitivity to doxorubicin.	(65) (66),
	Esophageal squamous cell carcinoma	Significantly upregulated; drives progression	HIF-1α	Stabilized by METTL14-mediated m6A; deubiquitinates and stabilizes HIF-1α → activates VEGF/GLUT1.	METTL14-OTUD7B-HIF-1α axis drives malignancy; high expression predicts poor survival.	(10, 71)
	Hepatocellular carcinoma	Downregulated; tumor-suppressive	p53	Directly deubiquitinates and stabilizes p53 → induces apoptosis and inhibits tumor growth.	Expression positively correlates with p53 level; overexpression suppresses tumor growth *in vivo*.	(76, 77)
Non-neoplastic Diseases	Experimental autoimmune encephalomyelitis	Upregulated in CNS	RIPK1, GFAP	1. Deubiquitinates RIPK1 to suppress NF-κB/MAPK and neuroinflammation. 2. Stabilizes GFAP and promotes glial scar formation.	Otud7b KO aggravates EAE; OTUD7B is neuroprotective.	(11)
	Pathological cardiac hypertrophy	Model-dependent dual role	HNF4α, SERCA2a	1. Pressure overload: stabilizes HNF4α → inhibits ferroptosis → protective. 2. Neurohormonal stress: deubiquitinates SERCA2a → disrupts Ca^2+^ homeostasis → pro-hypertrophic.	Opposing phenotypes in different models, showing strong context dependency.	(91, 92)

### 3.1 Neoplastic diseases

#### 3.1.1 Non-small cell lung cancer

NSCLC accounts for 85%-90% of all lung cancer cases and is the leading cause of cancer death globally. Its high mortality is mainly attributed to early lymph node metastasis and recurrence/resistance after treatment (29, 30). Although targeted therapies and immune checkpoint inhibitors have improved prognosis for some patients, elucidating the key molecular mechanisms driving angiogenesis and immune escape remains critical to breaking through therapeutic bottlenecks (31, 32). OTUD7B is significantly overexpressed in NSCLC, particularly in lung squamous cell carcinoma and adenocarcinoma, and its expression level is closely related to aggressive disease characteristics (7).

Mechanistically, OTUD7B drives lung cancer progression by activating multiple key signaling pathways. First, it promotes tumor cell proliferation and survival by stabilizing EGFR and enhancing phosphorylation activation of its downstream Akt/mTOR pathway. Second, OTUD7B expression is significantly positively correlated with vascular endothelial growth factor (VEGF) levels; functionally, it upregulates VEGF secretion, effectively promoting endothelial cell angiogenesis to provide nutritional support for tumor growth (7). Furthermore, studies reveal a negative feedback regulatory relationship between OTUD7B and the tumor-suppressive microRNA miR-491-5p. miR-491-5p directly inhibits OTUD7B expression, while OTUD7B deubiquitinates and stabilizes VEGFA protein. When miR-491-5p is downregulated, its inhibition of OTUD7B is relieved, leading to VEGFA accumulation and subsequently promoting tumor vasculogenic mimicry (9). OTUD7B also acts as a deubiquitinase that stabilizes TRAF3 to inhibit the activity of NIK, a kinase in the non-canonical NF-κB pathway; this function can inhibit drug-induced tumor invasion and metastasis under specific conditions (e.g., when using the Smac mimetic LCL161) (4, 33, 34).

Functionally, *in vitro* experiments confirm that OTUD7B overexpression significantly enhances the proliferation, migration, and invasion capabilities of lung cancer cells. *In vivo* studies corroborate this, showing that OTUD7B overexpression effectively promotes tumor growth in nude mouse xenograft models (7). Meanwhile, administration of an agonist (agomir) that upregulates miR-491-5p demonstrates anti-tumor effects *in vivo* by inhibiting the OTUD7B/VEGFA axis (9). Additionally, overexpression of wild-type OTUD7B (but not enzymatically inactive mutants) inhibits LCL161-induced lung metastasis *in vivo* (4). Evidence from both cell and animal models collectively supports the role of OTUD7B as a key pro-oncogenic molecule in NSCLC.

Collectively, in NSCLC, OTUD7B forms a primarily oncogenic network via EGFR/Akt/mTOR, miR-491-5p/VEGFA, and TRAF3/NIK axes, inhibiting metastasis only under specific drug treatments, and acts as a critical driver of lung cancer progression.

#### 3.1.2 Pancreatic cancer

Known as the “King of Cancers,” pancreatic cancer is renowned for its extremely high mortality rate and lack of effective treatments (35). As one of the most malignant tumors, its high fatality rate ranks it among the top causes of cancer-related deaths globally (36). Due to the absence of biomarkers that can specifically indicate the disease at an early stage, approximately 80% of patients are diagnosed with distant metastasis, missing the opportunity for radical surgery (37). Moreover, these patients generally exhibit resistance to existing radiotherapy and chemotherapy regimens, resulting in a five-year overall survival rate consistently below 10% (38, 39).

In the malignant progression of pancreatic cancer, OTUD7B is confirmed as a key effector molecule. It drives tumor cell proliferation, migration, and invasion by mediating the stabilization of the Notch1 intracellular domain (N1ICD) via the transmembrane serine protease RHBDL2, thereby activating the Notch signaling pathway (1) (40–42). Specifically, RHBDL2 first cleaves the Notch1 receptor via its protease activity to release the transcriptionally active N1ICD fragment; subsequently, RHBDL2 recruits OTUD7B to this complex to jointly regulate N1ICD stability (1, 42). As a deubiquitinase (DUB), OTUD7B plays a core catalytic role in this process: it specifically removes ubiquitin chains from N1ICD, thereby inhibiting its degradation via the ubiquitin-proteasome pathway. Experimental evidence indicates that knocking down OTUD7B leads to a significant increase in N1ICD ubiquitination levels and decreased protein stability, whereas overexpressing wild-type OTUD7B effectively reduces N1ICD ubiquitination and enhances its stability. Notably, when using catalytically inactive mutants (e.g., C194S), OTUD7B completely loses the ability to stabilize N1ICD, indicating that its deubiquitinase activity is essential for this function (43).

It is important to note that RHBDL2 itself lacks deubiquitinase activity; its stabilizing effect on N1ICD relies entirely on functional collaboration with OTUD7B. Once OTUD7B expression or enzymatic activity is inhibited, RHBDL2-mediated N1ICD accumulation and its pro-oncogenic effects (including cell proliferation, migration, and invasion) can be significantly reversed (44, 45). Stabilized N1ICD enters the nucleus to activate the transcription of multiple key downstream target genes of the Notch signaling pathway, including HES1, HEY1, ZEB1, MMP9, SNAIL1, and TWIST1. These genes are widely involved in regulating cell cycle progression, epithelial-mesenchymal transition (EMT), and the formation of invasive phenotypes, thereby directly driving the malignant biological behaviors of pancreatic cancer cells (43, 45, 46).

In conclusion, the OTUD7B-RHBDL2-N1ICD axis constitutes a functionally tight and mechanistically clear oncogenic signaling pathway in pancreatic cancer, revealing a new mechanism for abnormal activation of the Notch pathway and providing potential intervention nodes for targeted therapy.

#### 3.1.3 Gastric cancer

Gastric cancer is a high-incidence malignant tumor of the digestive tract globally and the third leading cause of cancer-related death. It is characterized by strong invasiveness, difficulty in early diagnosis, and frequent severe lymphatic metastasis (47–49). In the progression of gastric cancer, OTUD7B acts as a clear oncogenic driver: clinical samples show that OTUD7B is significantly overexpressed in gastric cancer tissues, and high levels are positively correlated with poor patient prognosis and tumor progression (50).

The core mechanism by which OTUD7B drives gastric cancer progression involves multi-pathway synergistic regulation. On one hand, it acts by directly targeting YAP1, a key effector of the Hippo signaling pathway: as a deubiquitinase, OTUD7B specifically removes ubiquitin chains from YAP1, preventing its recognition and degradation by E3 ligases (such as β-TrCP). This significantly extends the half-life of YAP1 and promotes its nuclear translocation. Once in the nucleus, YAP1, acting as a transcriptional co-activator, strongly initiates the expression of the downstream target gene NUAK2 (an AMPK-related kinase), which in turn promotes gastric cancer cell proliferation, colony formation ability, and tumorigenicity *in vivo*. Functional experiments confirm that knocking down OTUD7B leads to a rapid decline in YAP1 protein levels and inhibits NUAK2 expression, arresting malignant phenotypes; conversely, re-expression of YAP1 reverses the growth inhibition caused by OTUD7B deficiency, establishing the OTUD7B-YAP1-NUAK2 axis as a key signaling cascade driving gastric cancer progression (20).

On the other hand, OTUD7B promotes gastric cancer metastasis by activating the WNT/β-catenin signaling pathway. It interacts with the UBA and HMG domains of LEF1, promoting LEF1 nuclear localization and enhancing its binding with β-catenin in the nucleus, significantly upregulating approximately 75% of Wnt target genes and strengthening Wnt signal output. Simultaneously, OTUD7B activates downstream target genes (such as NUAK2, Snail, Slug, CDK6, CTGF, and BIRC5) by deubiquitinating and stabilizing YAP1 protein, synergistically promoting gastric cancer cell proliferation and metastasis (50). Notably, abnormal activation of the Wnt/β-catenin pathway not only drives EMT and acquisition of stemness but is also closely related to multidrug resistance (51). Clinical sample analysis shows that high β-catenin expression correlates with reduced CD8+ T cell infiltration in the gastric cancer microenvironment; inhibiting this pathway can enhance sensitivity to PD-1 antibody therapy, suggesting that the OTUD7B-Wnt axis may also participate in constructing an immunosuppressive microenvironment (52, 53).

In summary, in gastric cancer, OTUD7B promotes malignant tumor progression through a “dual-pathway synergy” mechanism (YAP1/NUAK2 axis driving proliferation, WNT/β-catenin axis promoting metastasis) while participating in the formation of an immunosuppressive microenvironment. Its high expression is closely related to poor patient prognosis and therapeutic resistance. This finding provides a key molecular basis for precise classification and targeted therapy of gastric cancer (e.g., interventions targeting the OTUD7B-YAP1 or OTUD7B-Wnt axes).

#### 3.1.4 Breast cancer

Breast cancer is the most common malignant tumor in women, with approximately 70% of cases expressing estrogen receptor alpha (ERα), making ERα a key target for endocrine therapy (54). However, clinical challenges remain severe, with 30%-50% of patients experiencing therapeutic resistance and recurrence, while distant metastasis is the primary cause of death (55). In this complex disease context, the deubiquitinase OTUD7B plays a key “dual pro-oncogenic” role, with mechanisms exhibiting significant subtype specificity, driving proliferation, metastasis, and stemness maintenance in different breast cancer subtypes by stabilizing key proteins such as ERα, LSD1, and FOXM1 (15, 17, 34).

Regarding mechanisms, in ERα-positive breast cancer, high expression of OTUD7B is positively correlated with ERα protein levels and is an independent risk factor for poor patient prognosis (17). The core mechanism lies in OTUD7B’s ability to directly interact with the AF1 domain of ERα, specifically removing K11 and K48-linked ubiquitin chains from ERα, thereby inhibiting its degradation via the proteasome pathway and significantly extending the ERα protein half-life (56). This stabilization leads to continuous transcriptional activation of downstream target genes (such as PS2, PDZK1, GREB1), promoting cell transition from G1 to S phase and accelerating cell proliferation and migration.

In basal-like breast cancer, OTUD7B strongly promotes tumor metastasis by regulating the LSD1 pathway. Mechanistically, OTUD7B specifically removes K63-linked ubiquitin chains from lysine residues 226 and 277 of the histone demethylase LSD1, thereby stabilizing LSD1 protein and maintaining the integrity of its core repressor complex with CoREST/HDACs (34). This complex regulates histone modification levels (such as H3K4me2 and H3K9me2) through genome-wide occupancy, thereby influencing the transcription of cell cycle-related genes (CyclinD1, CDK6) and key metastasis genes (Snail). If OTUD7B is deficient, LSD1 is rapidly degraded by the proteasome via a p62-mediated pathway, leading to disassembly of the core complex and abnormally elevated histone methylation levels, ultimately significantly inhibiting the migration, invasion, and lung metastasis capabilities of breast cancer cells (15).

Additionally, OTUD7B plays a key role in maintaining breast cancer stem cell (BCSC) stemness. In various cell lines such as MDA-MB-468, MDA-MB-453, and MCF7, OTUD7B directly interacts with the transcription factor FOXM1, reducing the polyubiquitination level of FOXM1 through deubiquitination modification and extending its protein half-life. Silencing OTUD7B leads to accelerated degradation of FOXM1, subsequently downregulating the expression of stemness markers such as Sox2, Nanog, CD44, and EpCAM, significantly weakening tumor sphere formation ability and cell proliferation capacity; conversely, overexpression of FOXM1 completely reverses these inhibitory effects caused by OTUD7B deficiency (34).

In conclusion, existing research confirms OTUD7B as a multifunctional driver in breast cancer progression: it promotes proliferation by stabilizing ERα in ERα-positive subtypes, drives metastasis by stabilizing LSD1 in basal-like subtypes, and maintains tumor stemness by stabilizing FOXM1 across various subtypes. This broad substrate specificity makes it a key therapeutic target spanning different molecular subtypes of breast cancer.

#### 3.1.5 Peripheral T-cell lymphoma

Peripheral T-cell lymphoma (PTCL) is a group of highly heterogeneous malignant tumors originating from mature T cells, characterized by strong aggressiveness, poor prognosis, and easy recurrence. PTCL represents a group of rare, highly heterogeneous, and typically aggressive non-Hodgkin lymphomas (NHL) originating from mature T cells or natural killer (NK) cells, accounting for approximately 10% to 15% of all NHL cases (57, 58). Due to the lack of specific driver mutation targets, current chemotherapy regimens have limited efficacy for many subtypes, making it urgent to deeply analyze key signaling nodes regulating T cell survival and proliferation (59).

OTUD7B acts as a key “oncogenic survival factor” in the occurrence and development of PTCL, with its high expression significantly positively correlated with poor patient prognosis (60). Mechanistic studies indicate that OTUD7B promotes the malignant phenotype of lymphoma cells through multiple parallel pathways: First, it directly binds and stabilizes the transcription factor LEF1 (a key effector of the Wnt pathway) by removing its K48 ubiquitination modification to prevent proteasomal degradation, thereby continuously activating the Wnt/β-catenin signaling axis, driving the expression of cell cycle proteins (such as CyclinD1) and promoting cell proliferation (1). Second, OTUD7B can stabilize the anti-apoptotic protein Mcl-1 or inhibit the activity of pro-apoptotic factors through deubiquitination modification, conferring strong resistance to chemotherapy drug-induced apoptosis in lymphoma cells. Additionally, it may maintain the undifferentiated state and stemness characteristics of T cells by regulating the Notch signaling pathway (stabilizing the Notch1 intracellular domain) (3). In summary, OTUD7B is a key oncogene driving PTCL progression by constructing a dual protective network of “proliferation-anti-apoptosis.” It not only promotes infinite tumor cell proliferation but also builds a molecular barrier against chemoresistance. Targeted inhibition of OTUD7B enzymatic activity holds promise as a new breakthrough to break the deadlock in PTCL treatment.

#### 3.1.6 Diffuse large B-cell lymphoma

Diffuse Large B-cell Lymphoma (DLBCL) is the most common aggressive non-Hodgkin lymphoma (NHL) in adults, accounting for 30%-40% of all NHL cases and up to 54%-65% of B-cell lymphomas (61, 62). Originating from mature B lymphocytes, the disease is highly heterogeneous, with significant differences in clinical presentation, molecular characteristics, genetic background, and treatment response (63, 64). In this clinical context, the deubiquitinase OTUD7B is confirmed to play a key tumor-suppressive role, with its expression level closely related to patient survival outcomes (65).

At the molecular mechanism level, OTUD7B primarily stabilizes the TRAF3 protein through deubiquitination modification, preventing its proteasomal degradation. This promotes the ubiquitination and clearance of NIK, effectively blocking the abnormal activation of the non-canonical NF-κB pathway (66). This cascade reaction significantly inhibits the secretion of pro-inflammatory and pro-metastatic factors such as IL-2 and MMP-9, thereby curbing malignant tumor progression. More critically, the expression level of OTUD7B directly regulates the sensitivity of DLBCL cells to chemotherapy drugs: overexpression of OTUD7B enhances cell sensitivity to doxorubicin, while silencing the gene induces a resistant phenotype (65).

#### 3.1.7 Esophageal squamous cell carcinoma

Esophageal squamous cell carcinoma (ESCC) is the main subtype of esophageal cancer, accounting for approximately 80%-90% of global cases. It is particularly prevalent in China and other Asian countries as well as some developing nations, with China contributing about 54% of global new cases. The disease mostly affects the middle and upper segments of the esophagus and differs significantly from esophageal adenocarcinoma in etiology, location, pathology, and treatment options, generally presenting characteristics of high incidence, high mortality, and poor prognosis (67–70). In this disease context, the deubiquitinase OTUD7B is identified as a key oncogene driving tumor progression, showing significant overexpression in ESCC tissues.

Studies indicate that OTUD7B expression is precisely regulated by epigenetic modifications: the methyltransferase METTL14 can specifically recognize and bind to the 2762nd site of OTUD7B mRNA, enhancing mRNA stability by mediating m6A methylation modification, thereby significantly upregulating OTUD7B protein expression levels (10, 71). This finding reveals the core regulatory status of the “METTL14-m6A-OTUD7B” axis in the occurrence and development of ESCC.

In terms of molecular mechanisms and functional effects, highly expressed OTUD7B specifically stabilizes the hypoxia-inducible factor HIF-1α through its deubiquitinase activity, preventing its proteasomal degradation. Stabilized HIF-1α then activates the transcription of key downstream target genes such as vascular endothelial growth factor (VEGF) and glucose transporter 1 (GLUT1), strongly promoting tumor cell proliferation, invasion capability, and adaptation to the hypoxic microenvironment (72). Functional experiments further confirm the importance of this pathway: in ESCC cell lines such as TE-8 and TE-1, silencing METTL14 significantly reduces the m6A modification level and mRNA stability of OTUD7B, thereby inhibiting malignant biological behaviors of the cells; conversely, overexpression of OTUD7B effectively reverses the inhibitory effects induced by METTL14 knockdown. These results not only elucidate the specific molecular pathway by which OTUD7B promotes ESCC progression but also suggest that targeting this axis may become a potential strategy for treating esophageal squamous cell carcinoma (10, 73).

#### 3.1.8 Hepatocellular carcinoma

Hepatocellular carcinoma is a digestive tract tumor with high malignancy and extremely poor prognosis. Its occurrence and development are closely related to hepatitis B virus/hepatitis C virus infection, alcoholic liver disease, etc. Due to difficulties in early diagnosis, rapid progression, and susceptibility to vascular invasion and intrahepatic metastasis, the 5-year survival rate of patients is usually less than 15% (74, 75). In this severe clinical context, the deubiquitinase OTUD7B reveals a unique tumor-suppressive function, forming a sharp contrast to its pro-oncogenic roles observed in many other solid tumors.

Its core mechanism lies in OTUD7B being a direct deubiquitinase of the tumor suppressor protein p53. Studies show that OTUD7B can bind to p53 (including wild-type and common mutants such as R249S and Y220C) and, through its enzymatic activity, specifically remove polyubiquitin chains from the p53 protein. This effectively antagonizes p53 ubiquitination and degradation mediated by the E3 ubiquitin ligase Mdm2, significantly improving p53 protein stability. This function strictly depends on the C194 site of its catalytic center (function is lost in the C194S mutant) (76). Stabilized p53 then activates the mitochondrial apoptosis pathway, manifested by upregulating pro-apoptotic proteins PUMA and BAX, downregulating anti-apoptotic protein Bcl-2, and inducing cleavage of caspase-3 and PARP, ultimately inhibiting liver cancer cell proliferation and tumor growth. This effect is entirely dependent on the presence of p53 (77). *In vivo* experiments further confirm that overexpression of OTUD7B significantly inhibits the growth of xenograft tumors in nude mice (78). Additionally, p53 itself can act as a transcription factor to negatively feedback and inhibit OTUD7B transcription, forming a precise bidirectional regulatory loop between the two (76).

In summary, in hepatocellular carcinoma, OTUD7B exerts a key tumor-suppressive role by stabilizing p53 and inducing apoptosis. This provides a mechanistic explanation for the clinical association between its low expression and poor patient prognosis and lays a theoretical foundation for developing novel therapies for p53 wild-type or specific mutant-type HCC based on strategies to upregulate OTUD7B function.

### 3.2 Non-neoplastic diseases

#### 3.2.1 Experimental autoimmune encephalomyelitis

Experimental autoimmune encephalomyelitis (EAE) is a classic animal model of multiple sclerosis (MS), with core pathological features including abnormal astrocyte activation, central nervous system inflammatory infiltration, and demyelination (79). Chemokines secreted by astrocytes are key driving factors mediating immune cell infiltration into the central nervous system (80). Research finds that OTUD7B is highly expressed in spinal cord astrocytes of EAE mice and is significantly upregulated in both the core and marginal areas of inflammatory lesions (81–83).

OTUD7B indeed exerts neuroprotective effects in EAE through a dual mechanism:

First, inhibiting neuroinflammatory responses: In astrocytes, OTUD7B effectively inhibits excessive activation of TNF-induced NF-κB and MAPK signaling pathways by performing sequential K63 and K48 site deubiquitination modifications on receptor-interacting protein kinase 1 (RIPK1). This regulation significantly reduces the secretion of chemokines (such as CCL2, CXCL10), thereby decreasing the infiltration of pathogenic CD4+ T cells into the central nervous system (CNS) and alleviating neuroinflammation (11, 84, 85).

Second, promoting glial scar formation to limit inflammation spread: OTUD7B also stabilizes GFAP protein levels by performing K48 site deubiquitination modification on glial fibrillary acidic protein (GFAP), preventing its proteasomal degradation. Simultaneously, OTUD7B can enhance IL-6/STAT3 signaling pathway activity to promote GFAP mRNA transcription, further enhancing reactive astrogliosis. This enhanced reactive astrocyte population forms a glial scar, acting as a physical barrier to limit the spread of inflammation to surrounding healthy tissue, thus playing a protective role (11, 86). These findings reveal the protective role of OTUD7B in EAE, providing a new target for MS treatment.

#### 3.2.2 Pathological cardiac hypertrophy

Pathological cardiac hypertrophy refers to a maladaptive and adverse myocardial remodeling process that occurs in the heart under long-term pressure or volume overload, neurohormonal activation (e.g., angiotensin II, norepinephrine), genetic mutations (e.g., hypertrophic cardiomyopathy-related gene variants), or other pathological stimuli (87, 88). Unlike physiological cardiac hypertrophy (e.g., induced by exercise training), pathological cardiac hypertrophy is usually accompanied by structural and functional changes such as abnormal enlargement of cardiomyocytes, interstitial fibrosis, inflammatory infiltration, mitochondrial dysfunction, calcium homeostasis imbalance, enhanced oxidative stress, and sparse myocardial microvessels, eventually progressing to heart failure, arrhythmias, or even sudden death (89, 90).

The role of OTUD7B in pathological cardiac hypertrophy is model-dependent, presenting dual effects of protection and pro-hypertrophy. On one hand, in pressure overload-induced cardiac hypertrophy models, OTUD7B exerts a protective effect through its deubiquitinase activity. In this context, OTUD7B expression is downregulated; cardiac-specific knockout exacerbates cardiac hypertrophy, while overexpression alleviates the pathological phenotype (91). The mechanism involves OTUD7B specifically removing K48-linked ubiquitin chains from HNF4α, stabilizing the HNF4α protein, inhibiting its proteasomal degradation, thereby maintaining the expression of fatty acid oxidation (FAO)-related genes and inhibiting ferroptosis. The use of the ferroptosis inhibitor ferrostatin-1 can alleviate cardiac hypertrophy caused by OTUD7B deficiency, confirming the existence of this protective pathway (91). On the other hand, under other pathological conditions stimulated by neurohormones, OTUD7B exhibits a pro-hypertrophic effect. In this scenario, OTUD7B expression is elevated in pathological hypertrophic myocardium of humans and mice; cardiomyocyte-specific knockout alleviates cardiac hypertrophy, while overexpression aggravates the lesion (92). The mechanism involves OTUD7B removing K63-linked ubiquitin chains at the K628 site of SERCA2a, thereby enhancing the interaction between SERCA2a and phospholamban (PLN), interfering with Ca2+ handling in cardiomyocytes, and ultimately driving cardiomyocyte hypertrophic responses (92).

In summary, the function of OTUD7B in pathological cardiac hypertrophy presents strong context dependency, capable of exerting either protective effects or promoting disease progression, highlighting the complexity of its precise intervention.

### 3.3 Molecular determinants of OTUD7B functional plasticity across disease contexts

Currently available studies have demonstrated that OTUD7B exerts diametrically opposing roles across malignancies: it promotes tumor progression in non-small cell lung cancer, gastric cancer, breast cancer, and esophageal squamous cell carcinoma, yet suppresses tumor growth in hepatocellular carcinoma and diffuse large B-cell lymphoma. The following analysis aims to systematically explore the potential mechanisms underlying this functional plasticity, moving from phenomenological descriptions toward in-depth investigation of its causal regulatory determinants.

#### 3.3.1 Cell-type-specific substrate availability

The functional output of OTUD7B is not determined by altered intrinsic enzymology, but by differential substrate availability and scaffolding proteins across cellular contexts. In dendritic cells, OTUD7B stabilizes TRAF2 to promote canonical NF-κB signaling and CD8+ T cell priming (23); conversely, in B cells, it stabilizes TRAF3 to suppress non-canonical NF-κB activation (4). Similarly, in hepatocellular carcinoma harboring wild-type p53, OTUD7B binds and stabilizes p53 protein, antagonizing Mdm2-mediated ubiquitination and inducing mitochondrial apoptosis (60, 77, 93). This tumor-suppressive function is abrogated in p53-null or mutant backgrounds, where OTUD7B instead stabilizes epidermal growth factor receptor (EGFR) or Yes-associated protein 1 (YAP1), acquiring oncogenic properties. Thus, the mutational landscape of the tumor may dictate OTUD7B functional identity. In breast cancer, OTUD7B maintains lysine-specific demethylase 1 (LSD1)/CoREST complex integrity through a partner-switching mechanism; its deficiency triggers sequestosome 1 (p62)-mediated proteasomal degradation of LSD1, abrogating metastatic potential.

#### 3.3.2 Post-translational and redox modulation of enzymatic activity

Deubiquitinases are sensitive to post-translational modifications that modulate catalytic efficiency (94, 95). OTUD7B regulates focal adhesion kinase (FAK) phosphorylation status, thereby influencing extracellular signal-regulated kinase (ERK)/p38 mitogen-activated protein kinase (MAPK) signaling in cardiac fibroblasts (13). Although direct evidence for redox-mediated modification of OTUD7B catalytic cysteine remains to be experimentally demonstrated, the susceptibility of DUB catalytic cysteines to oxidative inactivation is well established. Such transient attenuation could explain intratumoral functional heterogeneity—preserving OTUD7B activity in normoxic tumor peripheries while suppressing it in hypoxic cores. Future studies employing site-specific redox proteomics are warranted to test this hypothesis.

#### 3.3.3 Microenvironmental signal integration

OTUD7B expression and activity are dynamically shaped by microenvironmental cues. In esophageal squamous cell carcinoma, methyltransferase-like 14 (METTL14)-mediated N6-methyladenosine (m6A) methylation stabilizes OTUD7B mRNA under hypoxic stress (10). In pressure overload-induced cardiac hypertrophy, OTUD7B protects against ferroptosis by stabilizing hepatocyte nuclear factor 4-alpha (HNF4α) and maintaining fatty acid oxidation (96). Conversely, under neurohormonal stimulation, elevated OTUD7B deubiquitinates sarcoplasmic/endoplasmic reticulum calcium ATPase 2a (SERCA2a) and disrupts calcium homeostasis, promoting hypertrophy (92). These model-dependent outcomes illustrate that identical molecular entities can produce diametrically opposed phenotypes depending on the prevailing pathophysiological stressor. Furthermore, in the tumor microenvironment, OTUD7B promotes vascular endothelial growth factor A (VEGFA)-dependent angiogenesis and vascular mimicry in non-small cell lung cancer (4, 97, 98), indirectly shaping immune cell infiltration patterns, though direct regulation of immune checkpoint molecules by OTUD7B remains uncharacterized.

#### 3.3.4 Genetic background and compensatory network rewiring

The functional consequence of OTUD7B modulation is further complicated by compensatory mechanisms. In hepatocellular carcinoma, p53 transcriptionally represses OTUD7B, forming a negative feedback loop that may limit the therapeutic window for OTUD7B-centric interventions (93, 99). These observations indicate that OTUD7B functions not in isolation but as a node within highly buffered regulatory networks.

#### 3.3.5 Methodological limitations and forward perspectives

Several caveats constrain current interpretations. First, the majority of functional evidence derives from cell line overexpression or knockdown models, which fail to recapitulate clonal heterogeneity and stromal complexity. Second, isogenic models systematically comparing p53-wildtype versus p53-mutant backgrounds in the same cancer type are lacking, precluding definitive causal attribution of substrate switching. Third, the absence of single-cell resolution datasets mapping OTUD7B expression to specific tumor subpopulations obscures whether its functional duality reflects cell-intrinsic programs or microenvironmental adaptation.

In summary, the context-dependent functional plasticity of OTUD7B arises from the integration of cell-type-specific substrate availability, post-translational enzymatic modulation, microenvironmental signal inputs, and genetic background-dependent network rewiring. Rather than representing an inherent molecular paradox, this plasticity reflects the sophisticated role of OTUD7B as a conditional signal integrator. Future research must move beyond binary oncogene or tumor-suppressor classifications and adopt multidimensional analytical frameworks to decode the precise contextual logic governing OTUD7B activity. These context-dependent outputs are schematically summarized in **Figure 3**.

## 4 Clinical translation applications of OTUD7B

### 4.1 Biomarker applications

In non-small cell lung cancer, the expression level of OTUD7B is an important prognostic indicator. Multiple studies confirm that in lung squamous cell carcinoma and adenocarcinoma, high expression is significantly associated with higher AJCC stages (Stage I-II vs. III-IV), lymph node and distant metastasis, and predicts poorer overall survival (**Table 2**). Multivariate regression analysis further confirms that OTUD7B is an independent risk factor for poor prognosis in lung squamous cell carcinoma patients (4, 7). Regarding drug sensitivity, cell lines with high OTUD7B expression show significantly higher IC50 values for the Smac mimetic LCL161 (12.5μM vs. 4.2μM, P<0.01), suggesting that its high expression can predict tolerance to this drug (7, 33).

**Table 2:** Overview of clinical translation and application of OTUD7B.

Application field	Specific application scenario/potential	Biological sample/model	Key findings/value	Proposed mechanism of action	References
Biomarker	Prognosis & drug response prediction in NSCLC	Tumor tissue (IHC/RNA-seq)	High expression linked to advanced stage, metastasis, shorter OS; predicts resistance to LCL161.	Stabilizes EGFR, VEGFA, TRAF3; activates AKT/VEGF; inhibits non-canonical NF-κB in specific contexts.	(4, 7, 33)
	Prognostic & efficacy biomarker in breast cancer	Tumor tissue, serum	High expression = shorter OS/RFS in ERα+; lower pCR to paclitaxel in TNBC.	Stabilizes ERα, LSD1, FOXM1; drives proliferation, metastasis, stemness.	(15) (17) (34),,
	Prognostic & chemosensitivity predictor in DLBCL	Tumor tissue, patient samples	High expression = better OS; chidamide upregulates OTUD7B → enhances chemo-sensitivity.	Stabilizes TRAF3 → inhibits non-canonical NF-κB; improves doxorubicin response.	(65, 66),
	Independent prognostic factor in gastric cancer	Tumor tissue	High expression = shorter DFS; correlates with high grade and lymphatic invasion.	Stabilizes YAP1, LEF1; activates YAP/NUAK2 and Wnt/β-catenin.	(50)
	Prognostic biomarker in ESCC	Tumor tissue	High expression = poorer OS; correlates with METTL14 and HIF-1α.	Stabilized by m6A; stabilizes HIF-1α → promotes hypoxia adaptation and angiogenesis.	(10, 71),
	Prognostic biomarker in HCC	Tumor tissue	Low expression = faster recurrence, shorter OS; correlates with p53 level.	Stabilizes p53 → induces apoptosis; acts as tumor suppressor.	(76, 77)
	Potential biomarker for heart failure	Myocardial tissue	OTUD7B downregulated in heart failure; correlates with LVEF and fibrosis.	Stabilizes HNF4α → inhibits ferroptosis; suppresses cardiac fibrosis.	(13, 91, 92)
	Potential biomarker for MS activity	MS lesion brain tissue	OTUD7B upregulated in lesions; correlates with EDSS score.	Stabilizes RIPK1, GFAP; suppresses neuroinflammation and promotes glial scar.	(11)
Therapeutic Target Development	Small molecule/peptide inhibitors	Pancreatic cancer, breast cancer models	Cys194 inhibitors suppress proliferation; peptide disruptors reverse tamoxifen resistance.	Inhibits OTUD7B catalytic activity; blocks substrate interaction → degrades oncoproteins.	(1, 17),
	Nucleic acid drugs (siRNA/miRNA mimic)	NSCLC, vascular remodeling models	siRNA inhibits NSCLC growth/metastasis; Sal-miR-1 inhibits vascular remodeling.	Silences OTUD7B; blocks oncogenic signaling.	(2, 9),
	Gene therapy (AAV9 overexpression)	Mouse myocardial infarction model	Cardiac-specific OTUD7B reduces fibrosis, improves LVEF, inhibits apoptosis.	Stabilizes cardioprotective substrates; inhibits fibrosis and apoptosis.	(13)
	Combination therapy strategy	DLBCL, tumor models	OTUD7B upregulation sensitizes to chemo; Wnt/β-catenin links to immune microenvironment.	Enhances chemosensitivity; modulates tumor immune microenvironment.	(50, 66),

In different subtypes of breast cancer, data present high heterogeneity. In the ERα-positive subtype, high OTUD7B expression is significantly associated with shorter overall survival (HR = 1.85, 95% CI: 1.21-2.83, P = 0.004) and relapse-free survival (HR = 1.72, P = 0.011) and is strongly positively correlated with ERα target genes PS2 (r=0.56, P<0.001) and PDZK1 (r=0.49, P = 0.002) (17, 34, 56). In basal-like breast cancer, copy number variation analysis shows an amplification frequency as high as 28%, and the lymph node metastasis rate in the amplification group is significantly higher than in the non-amplified group (65% vs. 32%, P = 0.009); those with high expression have significantly reduced distant metastasis-free survival (15). Furthermore, in triple-negative breast cancer (TNBC), the pathological complete response (pCR) rate to paclitaxel in the high-expression group is significantly lower than in the low-expression group (15.4% vs. 42.1%, P = 0.036), and *in vitro* knockdown of OTUD7B increases paclitaxel-induced apoptosis rates by 3.2-fold (P<0.001) (34).

In DLBCL, large-sample clinical analysis provides strong evidence of a protective effect: patients with high OTUD7B expression have significantly prolonged overall survival (OS) (Log-rank P = 0.021). More importantly, multivariate Cox regression analysis confirms it as an independent prognostic protective factor (HR = 2.359, 95% CI: 1.039-5.358, P = 0.04), meaning that after correcting for confounding factors such as age and stage, the high-expression group still has a significantly better risk-benefit profile. Conversely, low OTUD7B expression is significantly positively correlated with multiple extranodal lesions (P = 0.006) and high International Prognostic Index (IPI) scores (P = 0.022), suggesting its close association with disease aggressiveness (66). Additionally, chidamide treatment can significantly upregulate OTUD7B expression (P<0.01), and the high-expression group shows significantly enhanced sensitivity to the combination therapy of “chidamide + doxorubicin,” with cell apoptosis rates increasing by approximately 2.5-fold (67). Therefore, in DLBCL, the core translational value of OTUD7B lies in its dual function as a biomarker for prognostic stratification and treatment response prediction.

In gastric cancer, high OTUD7B expression is an independent poor prognostic factor; the 3-year disease-free survival rate of patients with high expression is significantly lower than that of the low-expression group (38.5% vs. 62.4%, P = 0.008). Multivariate Cox regression analysis further confirms that OTUD7B is an independent risk factor affecting the prognosis of gastric cancer patients (HR = 2.14, 95% CI: 1.15-3.98, P = 0.016). Moreover, elevated OTUD7B expression levels are significantly associated with more aggressive tumor characteristics, including high tumor grade (P = 0.003) and lymphatic invasion (P = 0.012) (93, 94).

In esophageal squamous cell carcinoma, high OTUD7B expression similarly indicates poorer overall survival (P = 0.019). Mechanistically, OTUD7B expression is strongly positively correlated with the protein levels of the RNA methylation regulator METTL14 (r=0.45, P<0.001) and the hypoxia-inducible factor HIF-1α (r=0.52, P<0.001), suggesting it may participate in the malignant progression of ESCC by regulating RNA epigenetic modifications or tumor hypoxia adaptation pathways (67).

In hepatocellular carcinoma, the expression pattern of OTUD7B presents significant tumor-suppressive characteristics, holding important clinical translation potential. Studies show that OTUD7B is underexpressed in HCC tissues, and its low expression is significantly associated with faster disease recurrence and shorter overall survival in patients, providing direct evidence for its role as a prognostic biomarker in HCC (76). Furthermore, OTUD7B expression levels are significantly positively correlated with tumor suppressor protein p53 levels in clinical samples, suggesting that detecting OTUD7B expression may indirectly reflect the stable state and functional activity of p53 protein within tumor cells (77). This association provides a new idea for molecular subtyping of HCC, helping to identify tumor subgroups where the p53 pathway function is preserved or potentially amenable to intervention via this pathway, thereby providing a basis for formulating precision treatment strategies.

In heart failure patients, OTUD7B mRNA levels in myocardial tissue are significantly decreased by 65% compared to normal controls (P<0.001). This reduction is not only statistically significant but also positively correlated with the core cardiac function indicator, left ventricular ejection fraction (LVEF) (r=0.68, P<0.001), indicating that higher OTUD7B expression correlates with better cardiac function. Simultaneously, OTUD7B expression levels are significantly negatively correlated with myocardial fibrosis area (r=-0.59, P = 0.002), suggesting it may play a protective role in inhibiting myocardial fibrosis. Additionally, in myocardial infarction animal models, the infarct area in the low OTUD7B expression group expanded by 1.8 times compared to the control group (P = 0.004), further supporting its key role in myocardial injury repair and protection (13, 91, 92).

In multiple sclerosis (MS), the expression pattern of OTUD7B presents an opposite trend: OTUD7B mRNA expression levels in lesion areas are 4.5 times higher than in normal white matter (P<0.001) and are positively correlated with Expanded Disability Status Scale (EDSS) scores (r=0.47, P = 0.013) (84, 85). This finding suggests that in inflammatory demyelinating diseases of the central nervous system, the upregulation of OTUD7B may be related to the degree of neurological impairment. Its specific mechanism may involve immune regulation or neuroglial cell activation, but further research is needed to clarify the causal relationship.

### 4.2 Therapeutic target development

The development of therapeutic strategies relies on precise quantitative data support, covering inhibitor half-maximal inhibitory concentration (IC50), percentage of phenotypic improvement after gene knockdown, combination index (CI) for combined medication, and optimal time windows for sequential therapy.

Existing research indicates that small molecule and peptide inhibitors targeting deubiquitinases (DUBs) show significant therapeutic potential in various cancer models, especially in pancreatic and breast cancers. In pancreatic cancer cell lines, a candidate small molecule inhibitor targeting the OTUD7B catalytic site Cys194 demonstrated potent anti-tumor activity. After 48 hours of treatment with this compound, cell viability significantly decreased by 70% (IC50 ≈ 2.5μM, P<0.001), while inducing efficient degradation of the NOTCH1 intracellular domain (N1ICD) protein, with a degradation rate as high as 85% (P<0.01) (1, 100, 101). This result indicates that covalent modification of the catalytic site cysteine residue can effectively block the deubiquitinase activity of OTUD7B, thereby affecting its downstream key signaling pathways (such as the NOTCH pathway) and exerting anti-proliferative effects.

In the field of breast cancer, particularly in ERα-positive subtypes, OTUD7B was found to stabilize estrogen receptor alpha (ERα) protein through deubiquitination, with its high expression associated with poor prognosis (17). Targeting this mechanism, researchers developed a peptide inhibitor specifically designed to disrupt the interaction between OTUD7B and ERα. In *in vitro* experiments, this inhibitor significantly reduced the colony formation ability of ERα-positive breast cancer cells by 60% (P = 0.003) and successfully reversed tamoxifen resistance, restoring the sensitivity of resistant cells to 80% of the level of parental cells (100–102). This suggests that interfering with the formation of the OTUD7B-ERα complex is a feasible strategy for overcoming endocrine therapy resistance.

Data on the *in vivo* efficacy of nucleic acid drugs and gene therapy are particularly prominent. Additionally, clinical sample analysis shows that OTUD7B and LSD1 are synchronously highly expressed in high-grade or metastatic breast cancer, with higher copy number amplification frequency in basal-like subtypes; basal-like breast cancer patients with high OTUD7B expression have significantly reduced distant metastasis-free survival (15). Experimental data indicate that silencing OTUD7B significantly reduces ERα levels and inhibits the proliferation and migration capabilities of MCF-7 and T47D cells, while overexpression of ERα can reverse this effect; *in vivo* studies further confirm that cholesterol-modified OTUD7B siRNA can effectively inhibit xenograft tumor growth, demonstrating clear *in vivo* therapeutic potential (34). Based on the fact that the histone deacetylase inhibitor chidamide can upregulate OTUD7B expression and produce synergistic anti-tumor effects with doxorubicin, this provides a new combination therapy idea for overcoming chemotherapy resistance in DLBCL by targeting OTUD7B (65).

In preclinical models of various diseases, intervention strategies targeting OTUD7B have shown significant therapeutic potential. In NSCLC mouse models, tail vein delivery of cholesterol-modified OTUD7B siRNA (2 mg/kg, twice weekly for 4 weeks) effectively inhibited tumor progression, manifested by a 75% reduction in tumor volume (P<0.001) and an 82% reduction in the number of lung metastatic nodules (P = 0.002), without causing obvious hepatotoxicity, suggesting that this targeted delivery system has good anti-tumor activity and safety (103). Its mechanism of action is closely related to the angiogenesis pathway regulated by OTUD7B: studies show that in NSCLC, the tumor-suppressive miR-491-5p can regulate the ubiquitination and stability of VEGFA by directly targeting OTUD7B mRNA, ultimately affecting tumor vasculogenic mimicry, cell proliferation, and migration processes, further establishing OTUD7B as a potential target for anti-angiogenic therapy. Furthermore, in vascular remodeling disease models, oral administration of the plant-derived Sal-miR-1 mimic (10 mg/kg/day for 2 consecutive weeks) reduced the neointimal area of injured vessels by 55% (P = 0.006) by inhibiting OTUD7B expression and restored the expression of the vascular smooth muscle cell contractile phenotype marker SM22α to 2.3 times that of the control group, indicating that this intervention can inhibit pathological vascular remodeling by restoring the functional phenotype of vascular smooth muscle cells (2, 103). These research results collectively reveal the key regulatory role of OTUD7B in tumors and vascular diseases and provide preclinical evidence for developing RNA interference or miRNA mimic therapies based on this target.

Additionally, in myocardial infarction (MI) mouse models, cardiac-specific overexpression of OTUD7B via AAV9 vector (injection dose of 1x10^11 viral genomes) showed the following results after 4 weeks: cardiac fibrosis area reduced by 40% (P = 0.008), left ventricular ejection fraction (LVEF) improved by 15% (P = 0.012), and cardiomyocyte apoptosis index decreased by 65%. This indicates that OTUD7B has significant cardioprotective effects post-MI, effectively reducing fibrosis, improving cardiac function, and inhibiting cardiomyocyte apoptosis (13, 91, 92).

### 4.3 Feasibility assessment and technical bottlenecks of OTUD7B-targeted therapy

While the preclinical strategies outlined above demonstrate the modifiability of OTUD7B activity, several technical bottlenecks constrain their clinical translation. The foremost challenge is catalytic site selectivity. The OTU domain is highly conserved across family members; OTUB1 likewise relies on an active-site cysteine to execute its non-canonical ubiquitin transfer blockade and maintain PD-L1 stability (21). Broad-spectrum catalytic inhibitors risk off-target suppression of OTUB1, which could paradoxically enhance immune checkpoint signaling and compromise antitumor efficacy (21).

Non-catalytic approaches, such as peptide disruptors of the OTUD7B-ERα interaction, circumvent the selectivity issue but face distinct pharmaceutical limitations. Protein-protein interaction surfaces typically lack deep binding pockets amenable to high-affinity small-molecule engagement, resulting in suboptimal pharmacokinetic profiles. PROTAC technology offers an alternative degradation paradigm (26–28). However, the absence of OTUD7B-specific ligands currently restricts its application. Precedents for selective protein degradation support technical feasibility, yet extension to OTU family members remains unrealized. Furthermore, novel design strategies to enhance PROTAC efficiency continue to evolve.

Systemic toxicity constitutes a fundamental barrier. OTUD7B regulates NF-κB in immune homeostasis, p53 in DNA damage responses, and HNF4α in cardiac fatty acid oxidation (91, 96). Systemic inhibition therefore carries substantial risks of immunosuppression, genomic instability, or cardiotoxicity. Tumor-selective delivery systems—such as hypoxia-activated nanoparticles or antibody-drug conjugates—are prerequisite to distinguish pathological from physiological contexts. Resistance mechanisms further complicate therapeutic durability. Compensatory upregulation of alternative deubiquitinases, including USP7 for p53 stabilization and OTUB1 for ERα maintenance, may bypass OTUD7B inhibition. Biomarker-driven combination regimens and patient stratification will be essential to mitigate this adaptive rewiring.

Finally, the translational gap between preclinical models and clinical efficacy remains pronounced. Current evidence derives predominantly from cell line overexpression systems and murine xenografts. Validation in patient-derived organoids, immune co-culture platforms, and humanized mouse models is largely absent, creating uncertainty regarding the behavior of OTUD7B-targeted agents in human disease microenvironments.

## 5 Summary and future perspectives

OTUD7B (Cezanne) is a key member of the OTU deubiquitinase family, playing an important regulatory role in various pathophysiological processes including tumors, immunity, and nervous system diseases. Through its OTU catalytic domain, UBA domain, and zinc finger domain, it constitutes a precise signal processing module capable of specifically recognizing and cleaving different types of ubiquitin chains such as K11, K48, and K63. The core function of OTUD7B lies in acting as a molecular switch for cellular signaling pathways. By regulating the ubiquitination modification levels of key substrates such as TRAF2/3, YAP1, β-catenin/LEF1, Sox2, LSD1, ERα, and p53, it profoundly affects the activity of core pathways including NF-κB, Wnt/β-catenin, Hippo, Notch, mTORC2, and p53.In tumor progression, the function of OTUD7B exhibits significant context dependency. In most solid tumors (such as NSCLC and gastric cancer), it drives malignant behavior by activating pro-survival and pro-metastatic signals (e.g., NF-κB, Wnt pathways) or stabilizing oncoproteins (e.g., YAP1, ERα), correlating with poor prognosis. However, in specific diseases (such as diffuse large B-cell lymphoma and hepatocellular carcinoma), it exerts clear tumor-suppressive effects by stabilizing TRAF3 and p53. Its function displays high situational dependency, serving as a key node molecule connecting cellular homeostasis and inflammatory responses, and participating in the balance of immune surveillance and escape. Its expression abnormalities are closely related to the occurrence and development of various diseases including cancer, autoimmune diseases, and neurodegenerative diseases.

However, translating basic research achievements of OTUD7B into clinical applications faces multiple core challenges. The primary challenge is that the mechanism of its functional heterogeneity remains unclear; the same molecule may regulate key pathways like NF-κB in completely opposite directions in different cell types and microenvironmental signals. Secondly, the spatiotemporal heterogeneity of the tumor microenvironment (TME) (such as hypoxia, acidosis) continuously interferes with the activity, substrate selection, and downstream signal thresholds of OTUD7B, making the efficacy of targeted intervention difficult to predict stably. Thirdly, pathways regulated by OTUD7B, such as NF-κB and p53, are crucial for maintaining normal immune homeostasis and tissue repair; systemic inhibition is prone to cause off-target toxicity. Therefore, developing intervention strategies that can precisely distinguish between pathological and physiological states is a fundamental difficulty. Finally, existing research models struggle to fully simulate the complexity of the human disease microenvironment, leading to a translational gap between preclinical data and clinical efficacy.

To overcome these challenges, future research should focus on three integrative directions. First, functional decoding needs to be achieved at the single-cell and spatial dimensions. Utilizing single-cell multi-omics and spatial transcriptomics technologies, we should systematically map the dynamic regulatory network of OTUD7B in various cell subpopulations within different disease microenvironments to elucidate the cellular and molecular basis of its functional heterogeneity. Secondly, efforts should be devoted to developing context-intelligent targeted tools, including exploring new intervention modalities (such as allosteric inhibitors, PPI blockers, or PROTAC degraders) and designing intelligent, cell-specific delivery systems (such as antibody-drug conjugates, microenvironment-responsive nanocarriers) to achieve precise strikes on diseased cells while minimizing systemic toxicity. Finally, it is imperative to construct a closed-loop translational research paradigm based on physiologically relevant models. Actively applying patient-derived organoids, immune co-culture systems, and humanized mouse models will allow for a more realistic evaluation of the efficacy and resistance mechanisms of strategies targeting OTUD7B (especially in combination with immunotherapy) and conduct biomarker-driven reverse translational research to accelerate the closed-loop transformation from clinical discovery to therapeutic application.

In summary, OTUD7B is a sophisticated signal dispatcher whose function is highly dependent on cellular and microenvironmental context. The key to future breakthroughs lies in precisely decoding its regulatory logic under specific pathological backgrounds and innovatively developing highly selective intervention tools and strategies based on this. Through interdisciplinary integration and the convergence of frontier technologies, systematically elucidating the complex biology of OTUD7B will not only hope to provide new diagnostic and therapeutic strategies for various refractory diseases such as cancer and autoimmune diseases but also contribute a key paradigm to the emerging therapeutic field of targeting deubiquitinases.

## Glossary

AAV9: Adeno-Associated Virus Serotype 9
AF1: Activation Function 1
AJCC: American Joint Committee on Cancer
APC/C: Anaphase-Promoting Complex/Cyclosome
BCSC: Breast Cancer Stem Cell
CH: Cardiac Hypertrophy
CI: Combination Index
CLOCK: Circadian Locomotor Output Cycles Kaput
CNS: Central Nervous System
DC: Dendritic Cell
DLBCL: Diffuse Large B-cell Lymphoma
DUB: Deubiquitinase
EAE: Experimental Autoimmune Encephalomyelitis
EDSS: Expanded Disability Status Scale
EMT: Epithelial-Mesenchymal Transition
ERα: Estrogen Receptor Alpha
ESCC: Esophageal Squamous Cell Carcinoma
FAO: Fatty Acid Oxidation
GFAP: Glial Fibrillary Acidic Protein
HCC: Hepatocellular Carcinoma
HIF-1α: Hypoxia-Inducible Factor 1-alpha
HR: Hazard Ratio
IPI: International Prognostic Index
K11/K48/K63: Lysine 11/48/63 (Ubiquitin chain linkages)
LEF1: Lymphoid Enhancer-Binding Factor 1
LSD1: Lysine-Specific Demethylase 1
LVEF: Left Ventricular Ejection Fraction
m6A: N6-methyladenosine
MAPK: Mitogen-Activated Protein Kinase
METTL14: Methyltransferase Like 14
MI: Myocardial Infarction
MS: Multiple Sclerosis
N1ICD: Notch1 Intracellular Domain
NF-κB: Nuclear Factor Kappa-Light-Chain-Enhancer of Activated B Cells
NHL: Non-Hodgkin Lymphoma
NIK: NF-κB-Inducing Kinase
NPC: Neural Progenitor Cell
NSCLC: Non-Small Cell Lung Cancer
OTU: Ovarian Tumor
OTUD7B: Ovarian Tumor Domain Containing 7B
pCR: Pathological Complete Response
PLN: Phospholamban
PTCL: Peripheral T-cell Lymphoma
RHBDL2: Rhomboid Protease 2
RIPK1: Receptor-Interacting Serine/Threonine-Protein Kinase 1
SERCA2a: Sarcoplasmic/Endoplasmic Reticulum Calcium ATPase 2a
Smac: Second Mitochondria-derived Activator of Caspases
TNBC: Triple-Negative Breast Cancer
TRAF2/3: TNF Receptor-Associated Factor 2/3
UPS: Ubiquitin-Proteasome System
UBA: Ubiquitin-Binding Domain
VEGFA: Vascular Endothelial Growth Factor A
VSMC: Vascular Smooth Muscle Cell
Wnt: Wingless-related integration site
YAP1: Yes-Associated Protein 1
ZF: Zinc Finger Domain.
